# Is it safe to receive kidneys from deceased kidney donors tested positive for covid-19 infection?

**DOI:** 10.1080/0886022X.2021.1931319

**Published:** 2021-06-30

**Authors:** Hatem Ali, Mahmoud Mohamed, Miklos Z. Molnar, Nithya Krishnan

**Affiliations:** a Renal Department, University Hospital Coventry & Warwickshire, Renal, Coventry, UK; b Renal Department, University Hospitals of Tennessee, Tennessee, USA; c Department of Nephrology and Hypertension, University of Utah, Salt Lake City, UT, USA

Dear Editor,

For kidney transplant being the ideal solution for patients with end-stage kidney disease, there is an increasing demand for donated organs available for transplantation. According to the recent United Network for Organ Sharing (UNOS) update, there are more than 110,000 candidates on the kidney transplant waitlist, with the number growing every year [1].

Our modern world is facing extraordinary circumstances while passing through a serious pandemic caused by the novel coronavirus (COVID-19) which may lead to multi-organ system failure & death [2]. 198,589 COVID-19 deaths were reported in the US on 19 September 2020, some of which may provide a potential source for kidneys available for transplantation [3]. In this report, we are discussing five kidney transplant patients who received kidneys from individuals who tested positive for the novel coronavirus. We aim to open a discussion into a new chapter of expanded-criteria kidney donation.

We retrospectively reviewed all renal transplant recipients registered in the UNOS database who had their transplants during the 1st wave of COVID-19 pandemic between 1st of March 2020 and 1st of December 2020. Patients who received kidney transplants from a deceased donor with a positive COVID-19 polymerase chain reaction (PCR) test within three months prior to transplant were included in our study.

The STARFILES used in our study were: KIDPAN, KIDPAN_IMMUNE_DISCHARGE, and DECEASED_DONOR_DATA. Patients were followed up till the 4th of December 2020. Data about recipient factors (age, sex, ethnicity, diabetes, hypertension, body mass index, cause of renal failure, number of previous transplants, date of renal transplant), transplant factors (type of induction therapy, maintenance immunosuppressive therapy, delayed graft functions, early post-operative rejection episodes, HLA mismatch, PRA level, cold ischemia time) and donor factors (age, sex, ethnicity, diabetes, hypertension, date of COVID-19 test, type of COVID-19 test) were collected. Outcome measured were patient and graft survival till the end of the follow-up.

We found five renal transplant patients received kidneys from three deceased donors who tested positive for COVID-19 infection using PCR test. Details for baseline characteristics for transplant recipients and donors are shown in Tables 1 and 2, respectively. The timing of the COVID-19 test ranged between one day and 55 days pre-transplant. All donors tested positive for COVID-19 infection using nucleic acid testing one day before the transplant operation. None of the transplant recipients had acute rejection episodes, graft, or patient loss till the time of follow-up.

**Table 1.** Baseline characteristics for transplant recipients.

	Recipient 1	Recipient 2	Recipient 3	Recipient 4	Recipient 5
Age	46	29	58	65	64
Sex	Male	Male	Male	Female	male
Ethnicity	White	Asian	White	White	White
Diabetes	No	No	No	No	yes
BMI kg/m^2^	30.34		28.79	33.11	35.01
Cause of renal failure	Polycystic kidney disease	Chronic glomerulonephritis-unspecified	Membranous nephropathy	unknown	IgA nephropathy
Number of previous transplants	0	0	0	0	0
Dialysis	yes	Yes	no	Missing data	Missing data
Date of renal transplant	06/08/2020	06/08/2020	25/09/2020	28/11/2020	28/11/2020
Induction therapy	Basiliximab	Basiliximab	Thymoglobulin	Missing	Missing
Maintenance immunosuppre-ssive therapy	Steroids, tacrolimus, mycophenolate mofetil	Steroids, tacrolimus, mycophenolate mofetil	Steroids, tacrolimus, Mycophenolate mofetil	Missing	Missing
HLA mismatch	3	4	5	1	3
PRA (%)	0	58	71	0	0
Cold ischemia time (hours)	26.03	19.3	16.75	Missing data	Missing data
Hepatitis C status	Negative	Negative	Negative	Negative	Negative
HIV status	Negative	Negative	Negative	Negative	Negative
Death	No	No	No	No	No
Delayed graft function	Yes	Yes	No	No	No
Acute post-operative rejection	No	No	No	No	No
Graft failure	No	No	No	No	No

BMI: Body Mass Index; PRA: Panel Reactive Antibodies.

**Table 2.** Baseline characteristics for donors.

	Donor 1 to recipient 1 and recipient 2	Donor 2 to recipient 3	Donor 3 to recipient 4 and recipient 5
Type of COVID-19 test	Nucleic acid test	Nucleic acid test	Nucleic acid test
Date of COVID-19 test	05/08/2020	01/08/2020	19/10/2020
Date if organ implantation			
Age	22	28	43
Sex	Male	Female	Male
Ethnicity	Hispanic	White	White
Diabetes Mellitus	no	no	no
CMV serology	Positive	Positive	Negative

The results of our study highlight the importance of considering deceased patients secondary to COVID-19 infection as potential kidney donors. Many uncertainties surround kidney donation during the COVID-19 era. The guidelines’ recommendations about kidney donation show disparities, low-quality evidence and are difficult to implement in clinical practice. The American Society of Transplant Surgeons recommends suspending all living donations unless necessary. The deceased donation can be resumed if it is in the interest of the recipient [4]. NHSB’s most recent guidelines recommend suspending organ donations from those who tested positive for the novel coronavirus. Moreover, it discourages donation from asymptomatic individuals who have been in a COVID-19-affected area in the last 28 days [5]. On the other hand, the American Society of Transplantation recommends that kidneys from donors with an intermediate risk of COVID-19 infection can be considered with caution. They suggested that it is safe to use organs from donors who are low-risk or COVID-19 negative, as well as those who have recovered from COVID-19 disease more than 28 days before donation [6].

Literature rejected the possibility of transmission of COVID-19 through blood and blood products of vertical transmission to the fetus from COVID-19 infected mothers [7]. Kwon et al. reported transfusion of blood and platelet to nine recipients from asymptomatic COVID-19-positive donors who donated at least three days before the onset of symptoms. No viral transmission was reported in the recipients [8]. Nevertheless, the data still limited to a small number of reported patients.

Utilizing kidneys from donors with infectious risks has the potential to become the standard of care in many centers [4–6]. The urge to minimize the number of candidates on the kidney transplant waitlist led to relax the criteria of accepting more marginal donors and an increasing utilization of medically complex kidney donors. In our series, we included deceased donors who tested positive for COVID-19 infection using PCR. It is imperative to consider false positivity when assessing PCR results. One of the methods to consider false positivity is to assess cycle threshold for PCR results. Unfortunately, we do not have the results for cycle threshold in our case series. However, we recommend that it should be discussed with local lab before considering transplantation [9]. It is also important to consider the vaccination status of the recipients. During the first wave of COVID-19 pandemic, none of the recipients were vaccinated. However, nowadays waitlist candidates should be vaccinated which increases the safety of using these donors.

Our report has certain limitations. Being retrospective in nature with the small number of patients included limits the ability to produce any causal interpretation. Another limitation is the short follow-up period. However, rejection episodes occur most often in the first six months after transplant. More studies are needed to address the short and long-term complications and outcomes for accepting kidneys from individuals who tested positive for the novel coronavirus. Additional work is urgently needed to clarify the post-donation health hazards among those patients and assure the safety of the patients. In conclusion, receiving kidneys from selected deceased donors who tested positive for COVID-19 infection may be safe and enable tapping in to a source of organ donation so far not considered.
